# The lymphedema patient experience within the healthcare system: a cross-sectional epidemiologic assessment

**DOI:** 10.1038/s41598-024-63145-1

**Published:** 2024-06-01

**Authors:** Catharine Bowman, Stanley G. Rockson

**Affiliations:** 1 Stanford University School of Medicine, Stanford, CA USA; 2 Department of Epidemiology and Population Health, Stanford University School of Medicine, Stanford, CA USA; 3 Stanford Center for Lymphatic and Venous Disorders, Stanford University School of Medicine, Falk Cardiovascular Research Center, 300 Pasteur Drive, Stanford, CA 94305 USA

**Keywords:** Lymphedema, Lymphoedema, Lymphatic care, Healthcare delivery model, Healthcare satisfaction, Lymphatic education, Quality of life, Epidemiology, Epidemiology, Outcomes research, Breast cancer, Cancer epidemiology, Gynaecological cancer, Head and neck cancer, Health care, Medical research

## Abstract

Lymphedema is a progressive lymphatic disease that potentiates physical and psychosocial distress. Despite its impact, patients reportedly encounter lymphatic ignorance throughout the healthcare system. This cross-sectional study aims to summarize clinical characteristics and interactions of lymphedema patients within the healthcare system. Two lymphedema patient cohorts were included: The Global Registry Analysis Cohort included lymphedema patients who contributed to an international digital lymphatic registry and the Interactions Cohort included patients who initiated a questionnaire about interactions with the medical system. The global registry was used to obtain demographic and clinical characteristics from affiliated lymphedema patients. A 23-item online questionnaire on healthcare experiences and satisfaction with lymphatic healthcare was then distributed to the Interactions Cohort. Complete responses were obtained from 2474 participants. Participants were a mean age of 57.5 ± 16.1 years and 51.4% had a cancer history. Participants reported substantial delays in diagnosis and treatment. Cancer-related and non-cancer-related lymphedema patients reported similar levels of perceived physician disinterest in their lymphedema; however, non-cancer-related lymphedema patients reported more care dissatisfaction. Ultimately, patients continue to face delays in lymphedema diagnosis and treatment. We developed an evidence-based model highlighting areas of reform needed to improve lymphatic education and healthcare.

## Introduction

Lymphedema is associated with substantial physical and psychosocial morbidity^1–3^. Lymphatic patients report a perceived lack of awareness and lymphatic ignorance when interacting with the medical system and public. Existing literature focuses on the impact of secondary cancer-related lymphedema on physical and psychosocial well-being through qualitative and smaller cohort studies. These studies have demonstrated that cancer-related lymphedema is associated with increased anxiety, depression, and diminished quality of life when compared to cancer survivors without lymphedema and with the general, non-cancer affected public^2,4,5^. Qualitative literature further elucidates these findings by exploring the unique challenges of lymphedema patients who are simultaneously journeying through the psychosocial complexities of cancer survivorship^1,6^. Patients also become subject to recurrent soft tissue infections that lead to re-hospitalization, causing additional psychosocial distress.

A common finding that has arisen from both qualitative and quantitative literature is the negative experiences of patients when interacting with the healthcare system. This observation reflects a reported disregard of lymphedema by clinical practitioners. For instance, Stolldorff et al.^7^ published a quote from a non-cancer-related lymphedema patient who stated, “I have discovered that most doctors are incredibly ignorant when it comes to lymphedema.” In some scenarios, patients have even received negative commentary from practitioners when seeking support for their chronic disease^4^. It is evident that many medical practitioners lack awareness of lymphatic disorders^8,9^. Despite compelling evidence from qualitative and smaller cohort-based studies on lymphedema patient interactions with the healthcare system, there is minimal epidemiologic documentation on the widespread impact of lymphedema and the associated healthcare interactions of patients. This study was designed to summarize the clinical characteristics and interactions of lymphedema patients within the medical system, and to conduct the first formal validation and quantitation of dissatisfaction with care in this patient community.

## Methods

### Study design

The Strengthening the Reporting of Observational Studies in Epidemiology checklist was used for this cross-sectional study (Supplementary information)^10^. The study was conducted at Stanford University. Participant recruitment and data collection occurred between June 2016 and July 2023. Two cohorts participated in this work, which included individuals with self-reported or practitioner-diagnosed lymphedema. The Global Registry Analysis (GRA) Cohort completed digital registry questions through the Lymphatic Education and Research Network (LE&RN) and the Interactions Cohort included patients affiliated with LE&RN who participated in an online questionnaire about interactions with the medical system. This study was overseen by the Stanford University Internal Review Board (protocol 64956) and North Star Review Board (protocol NB300093).

### Participants

#### GRA cohort

The LE&RN is the largest internationally recognized non-profit organization for lymphatic disease education, research, and advocacy^11^. In 2016, the LE&RN patient registry was established to collect clinical and demographic data on formally-diagnosed and self-identified adult and pediatric lymphatic patients from across the globe, comprising both those with lymphedema and other allied lymphatic pathologies^12^. Hence, participants were included in the analysis if they specifically had lymphedema. Because our recruitment efforts for this study were conducted through LE&RN, the LE&RN registry was used as a representative global sample to extract self-report demographic and clinical information to augment Interactions Cohort findings. Formal consent was not required for this portion of the study, as determined by the Stanford University Internal Review Board and North Star Review Board.

#### Interactions cohort

Participant enrollment began February 1st, 2022. A 23-item questionnaire was anonymously distributed through social media in partnership with LE&RN (Table 1). Participants were eligible if they were living with lymphedema and able to complete the online questionnaire. Participants were asked to anonymously report on their clinical characteristics as well as interactions with the healthcare system, based upon the pre-specified 23-item online questionnaire. Formal consent was not required for this portion of the study, as determined by the Stanford University Internal Review Board and North Star Review Board.

**Table 1:** outlines the items and answer options on the 23-item questionnaire.

Item	Answer options
Do you have lymphedema?	Yes
	No
Are you a cancer survivor?	Yes
	No (if no, please skip to question 14)
Did your cancer surgery include removal of lymph nodes?	Yes
	No
Did your cancer treatment include radiation?	Yes
	No
Did your health care provider(s) discuss lymphedema risk prior to surgery?	Yes
	No
	Not relevant
Did your health care provider(s) discuss lymphedema risk prior to surgery?	Yes
	No
	Not relevant
Were you given educational materials about lymphedema prior to cancer treatment?	Yes
	No
Before or during cancer treatment, I was given compression garments to use to reduce risk	Yes
	No
During cancer treatment, how often were you questioned about lymphedema symptoms?	Never
	Once
	Sometimes
	At each visit
During cancer treatment, how often were you examined to specifically look for lymphedema?	Never
	Once
	Sometimes
	At each visit
How often were you tested to detect early lymphedema during cancer treatment?	Never
	Once
	Twice
	Three times
	Four times or more
I was tested for lymphedema by	Measurements of my limbs by tape measure
	Perometry
	Bioimpedance (L-Dex or other)
	I never had a test
From start of cancer treatment to first symptoms of lymphedema required	0–1 month
	2–6 months
	7–12 months
	12–24 months
	More than two years
My lymphedema was diagnosed by:	My cancer surgeon
	My radiation oncologist
	My primary care physician
	Another specialist
	A doctor never made the diagnosis
My lymphedema was first observed by	Me
	A family member or friend
	My doctor or other health care professionals
From the time of first appearance of lymphedema symptoms, a diagnosis of lymphedema took	< 1 month
	1–3 months
	4–6 months
	7–12 months
	More than a year
Before the diagnosis of lymphedema was confirmed, I saw	One doctor
	Two doctors
	Three doctors
	Four doctors
	Five or more doctors
Have you been referred by a health care professional to a lymphedema therapist?	Yes
	No
	I referred myself
How long did it take from onset of lymphedema symptoms to start of treatment?	< 1 month
	1–3 months
	4–6 months
	7–12 months
	More than a year
With relationship to your quality of life and to your function, please rate the impact of your lymphedema diagnosis	Sliding Scale: No effect to Devastating
In your experience, how concerned have your doctors been about your lymphedema	Sliding Scale: Uninterested to Very Concerned
How would you describe your doctors in relationship to the diagnosis and treatment of lymphedema (please choose all that apply)?	My doctors were not able to make the diagnosis
	My doctors were able to diagnose lymphedema but were not able to recommend treatment
	My doctors knew a little bit about lymphedema treatment
	My doctors were very knowledgeable about lymphedema
With regard to the entire healthcare system, how satisfied are you with your lymphedema diagnosis and treatment	Sliding Scale: 100% Dissatisfied to 100% Satisfied

### Procedures and outcomes

#### GRA cohort

The LE&RN global patient registry consists of ~ 600 unique self-report items including demographic (e.g. sex assigned at birth—male/female), clinical (e.g. years since lymphedema diagnosis), treatment-related, and healthcare interaction-based questions. The registry included categorical and continuous items, and four sliding scale questions pertaining to distress, disease severity, and physician satisfaction. The registry opened in June 2016 and data extraction for this study occurred in July 2023. Given the known inconsistencies in the use of formal diagnostic criteria and coding for lymphedema in the medical community, self-report lymphedema status was used to minimize misclassification bias of the exposure.

#### Interactions cohort

A 23-item self-report questionnaire was developed based upon literature review and clinical expertise, and delivered via Qualtrics, a secure web-based platform intended for data collection^13^. The questionnaire focused on five domains: lymphedema status, clinical characteristics, diagnosis and treatment, quality of life, and interactions with the healthcare system. Two sliding scales were used to assess quality of life as it relates to lymphedema and level of satisfaction with lymphedema diagnosis/treatment. The questionnaire was delivered through Stanford University and remained opened from February 1st, 2022 to July 24th, 2023. In order to better address selection bias and generalizability, we used the LE&RN global patient registry as a representative subsample of the Interactions Cohort to obtain relevant demographic and clinical data.

### Statistical analysis

Statistical analyses were conducted using SAS OnDemand for Academics Version 3.81. A complete case analysis was undertaken after evaluation of missingness (less than five percent missingness identified across each primary variable). Statistical significance was defined as P < 0.05. A formal sample size calculation was not undertaken given its irrelevance to the methodology of this study, which was an outreach to the patient community to understand the breadth of experience across this population.

#### GRA cohort

Descriptive statistics were calculated for 85 registry items. Stratified analyses were undertaken for items that related to both non-cancer-related and cancer-related forms of lymphedema. Identification of non-cancer-related lymphedema did not attempt to discriminate between primary and secondary causes. Two-sided two-sample proportions tests and Wilcoxon Sum Rank tests were used to compare core demographic features between cancer-related and non-cancer-related lymphedema patients due to a lack of normality in the data distribution.

#### Interactions cohort

Descriptive statistics were calculated for all questionnaire items and lymphedema sub-group stratified analyses were undertaken, where applicable. Two-sided one-sample proportions tests were undertaken for whole-group items to compare proportions between categorical responses. Two-sided two-sample proportions tests were used to compare responses between cancer-related and non-cancer-related lymphedema participants. Sliding scale items were treated as continuous variables and Wilcoxon Sum Rank Tests were used to compare between cancer-related and non-cancer-related sub-groups due to a violation of the normality assumption.

## Results

At the completion of the investigation, 2326 participants had initiated the healthcare experiences questionnaire, and 1948 individuals completed it (83.8%). Of these participants, seven individuals identified as not having lymphedema and were excluded from the analysis. Hence, 1941 individuals were included in the final analysis. There were 539 individuals in the GRA Cohort who identified as having lymphedema or being unsure of their lymphedema diagnosis. The GRA questionnaire was completed by 533 of the 539 participants (98.9%).

### Demographics and clinical characteristics

The mean age of the LE&RN GRA Cohort was 57.5 ± 16.1 years and most participants were female (86.9%, Table 2). The mean age of the cancer-related lymphedema group was significantly greater than that of the non-cancer-related group (62.6 ± 12.6 years vs. 54.2 ± 17.3 years, P < 0.001), and there was a significant difference in the biological sex frequency distribution across groups (83.2% female in non-cancer-related group vs. 92.5% female in cancer-related group; P = 0.0038). The majority of participants (92.6%) were non-Hispanic or Latino and identified as White (87.9%). Although most participants were from the United States (89.2%), some participants were from Canada (3.5%), Australia (1.2%), and England (1.6%). The majority of participants held a post-graduate degree (32.3%) or bachelor’s degree (29.2%; P = 0.001). Approximately 18.8% of participants reported an average annual income of $50,000-$80,000 and had insurance (94.7%, Table 2). The income frequency distribution differed significantly between cancer-related and non-cancer-related subgroups (P = 0.0096). Furthermore, 36.6% reported using MedicAid or MediCare. Most participants reported annual lymphedema-related out-of-pocket expenses of < $500 or $1000–$5000. Eleven participants reported annual lymphedema-related costs of > $10,000. Of those who specified annual costs > $10,000, two participants provided qualitative comments on factors that were associated with these elevated costs. Factors included surgery and general lymphedema management. Although the majority of cancer-related (63.5%) and non-cancer-related (64.1%) patients did not change jobs due to lymphedema, approximately 60.6% modified work hours.

**Table 2:** demonstrates the demographic and clinical characteristics from the GRA Cohort.

	Non-cancer-related lymphedema patients (n = 323) [No. (%)]	Cancer-related lymphedema patients [(n = 206) No. (%)]	p-value^a^
Demographic characteristic			
Sex^b^			P = 0.0038
Male	50 (16.2)	15 (7.5)	
Female	257 (83.2)	185 (92.5)	
Decline to State	2 (0.6)	0 (0.0)	
Age (Mean [SD]), years	54.2 [17.3]	62.6 [12.6]	P < 0.0001
Time with Lymphedema (Mean ± [SD]), years	16.4 [16.0]	10.8 [7.5]	P < 0.0001
Race^c^			P = 0.3416
White	263 (86.2)	179 (90.4)	
Black or African American	17 (5.6)	2 (1.0)	
More than one race	13 (4.3)	7 (3.5)	
Asian	9 (3.0)	2 (1.0)	
American Indian/Alaska Native	1 (0.3)	0 (0.0)	
Decline to State	2 (0.7)	8 (4.0)	
Ethnicity^b^			P = 0.3338
Not Hispanic or Latino	287 (92.9)	184 (92.0)	
Hispanic or Latino	15 (4.9)	6 (3.0)	
Decline to State/Unknown	7 (2.3)	10 (5.0)	
Country^c^			P = 0.0011
United States	263 (85.1)	188 (95.4)	
Canada	14 (4.5)	4 (2.0)	
Australia	4 (1.3)	2 (1.0)	
England	8 (2.6)	0 (0.0)	
Ireland	3 (1.0)	1(0.5)	
France	2 (0.7)	0 (0.0)	
Other	15 (4.9)	2 (1.0)	
Education^c^			P = 0.1188
Less than 12 years of education	9 (2.9)	3 (1.5)	
High school diploma	52 (16.8)	31 (15.5)	
Associate’s degree	41 (13.3)	27 (13.5)	
Bachelor’s degree	87 (28.2)	62 (31.0)	
Post-graduate degree	93 (30.1)	71 (35.5)	
Other	27 (8.7)	6 (3.0)	
Income			P = 0.0096
< $10,000–$30,000	56 (17.3)	27 (13.1)	
$30,001–$50,000	46 (14.2)	22 (10.7)	
$50,001–$80,000	53 (16.4)	47 (22.8)	
$80,000–$120,000	54 (16.7)	32 (15.5)	
$120,001–$150,000	17 (5.3)	10 (4.9)	
> $150,000	39 (12.1)	45 (21.8)	
Prefer not to disclose	58 (18.0)	23 (11.2)	
Annual lymphedema expenses^c^			P = 0.6090
< $500	89 (28.8)	51 (25.6)	
$501–$1000	55 (17.8)	41 (20.6)	
$1001–$2000	63 (20.4)	42 (21.2)	
$2001–$5000	58 (18.8)	45 (22.6)	
$5001–$10,000	17 (5.5)	9 (4.5)	
> $10,000	8 (2.6)	3 (1.5)	
Prefer not to disclose	19 (6.1)	8 (4.0)	
Has health insurance^b^			P = 0.0778
Yes	288 (93.2)	193 (97.0)	
No	18 (5.8)	5 (2.5)	
Unsure	3 (1.0)	1 (0.5)	
Health insurance sub-groups^c^			P = 0.3807
Private commercial	78 (24.5)	65 (28.2)	
Prepaid health plan	122 (38.2)	85 (40.0)	
Medicare	88 (27.6)	73 (31.7)	
Medicaid	29 (9.1)	4 (1.7)	
Veteran affairs	2 (0.6)	3 (1.3)	
All lymphatic treatment covered by drug plan			P = 0.7340
Yes	63 (26.0)	49 (28.0)	
No	129 (53.3)	95 (54.3)	
Unsure	50 (20.7)	31 (17.7)	
Job change			P = 0.3786
Yes	103 (33.9)	64 (32.5)	
No	195 (64.1)	125 (63.5)	
Unsure	6 (2.0)	8 (4.1)	
Job hours change			P = 0.6408
Yes	182 (59.5)	123 (62.4)	
No	117 (38.2)	68 (34.5)	
Unsure	7 (2.3)	6 (3.1)	

Group percentage may not summate to 100% due to rounding.

Significance defined as P < 0.05.

^a^P-value based on Chi squared test for categorical variables and Student's t-test for continuous variables.

^b^ ‘Decline to state’ omitted from statistical analysis due to data sparsity.

^c^Categories collapsed for statistical analysis due to data sparsity. Race categories: “White”, “More than one race”, and “Black, African American, Asian or American Indian/Alaska Native”. Country categories: “United States”, “Canada”, and “Other”. Education categories: “High school diploma or less than 12 years of education”, “Associate’s degree”, “Bachelor’s degree”, “Post-graduate degree”, “Other”. Annual Lymphedema Expenses categories: “ < $500”, “$501–$1000”, “$1001-$2000”, “$2001–$5000”, “$5,001–$ > 20,000”, and “Prefer not to disclose”. Health Insurance Sub-Groups categories: “Private Commercial”, “Prepaid Health Plan”, and “Medicare, Medicaid, or Veteran Affairs”.

The majority of participants (75.4%) did not have a lymphatic diagnosis at birth. Most participants reported their lower extremities as the most common pattern of first presentation (75.5%), which was in keeping with the lower and upper limbs as the most common sites of current lymphatic disease (89.7%). The mean number of years lived with lymphedema in the cancer-related lymphedema group was shorter than that of non-cancer-related lymphedema patients (10.8 ± 7.5 years vs. 16.4 ± 16.0 years; Table 2, P < 0.0001). Sixty-seven participants reported co-morbid lipedema. Approximately 52.0% of participants had not experienced cellulitis; this occurred across both lymphedema sub-types. Of those participants who had cellulitis, 38.6% experienced greater than five episodes, yet 92.6% of the full subject cohort did not take prophylactic antibiotics.

### Healthcare interactions: cancer-related and non-cancer-related lymphedema patients

#### Diagnosis

**Figure 1:**
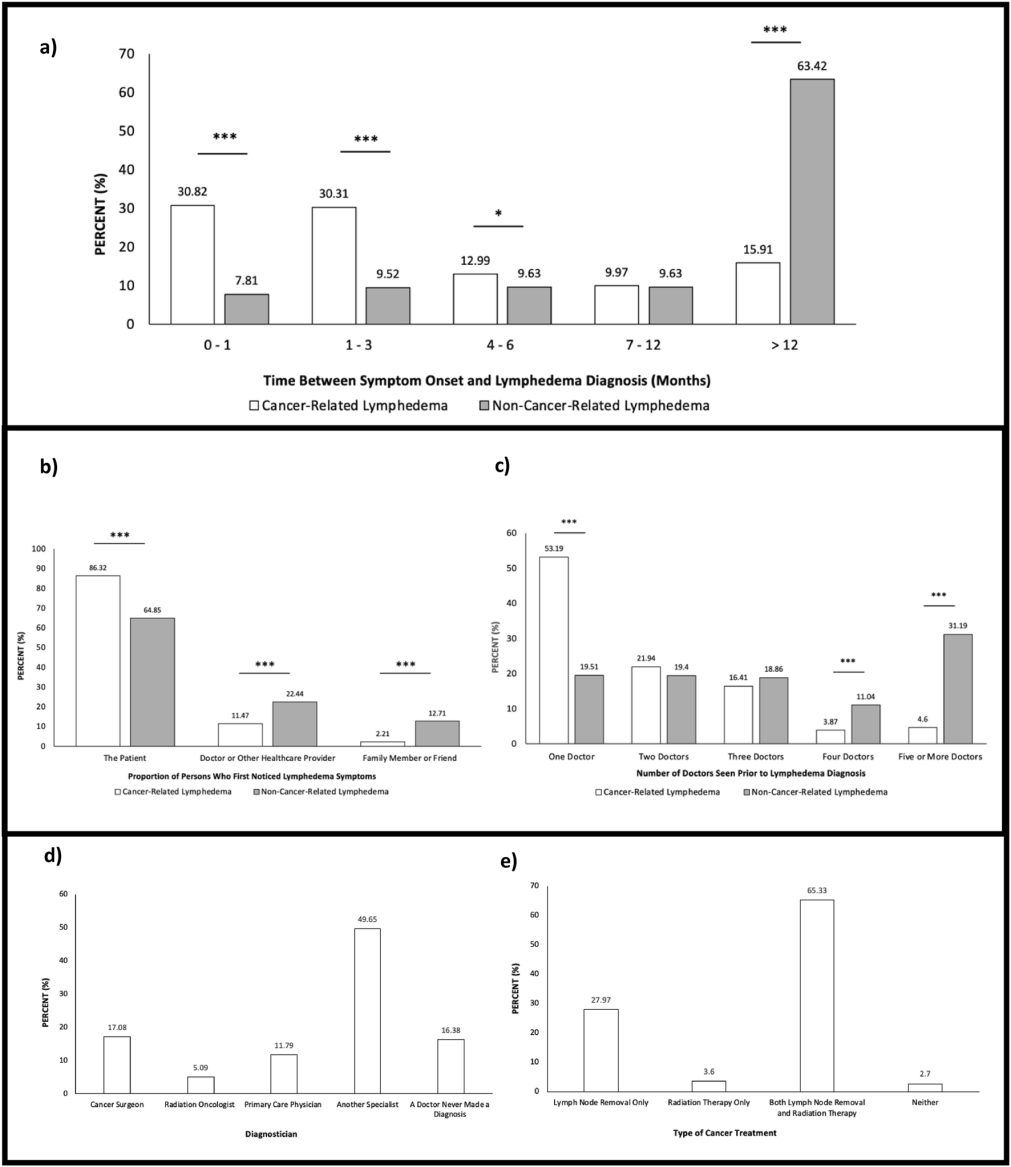
(**a**) Number of months between lymphedema symptom onset and lymphedema diagnosis, two-sample proportions test, *P < 0.05, ***P < 0.001 (n = 1936) (**b**) Distribution of who first noticed patients’ lymphedema, two-sample proportions test, ***P < 0.001 (n = 1930) (**c**) The number of physicians seen by patients prior to receiving lymphedema diagnosis, two-sample proportions test, ***P < 0.001 (n = 1890). (**d**) Distribution of practitioners who diagnosed participants’ lymphedema (n = 1001) (**e**) Proportion of cancer treatment modalities in cancer-related lymphedema cohort (n = 1001).

The majority of participants reported having a cancer history (51.4%). At the initial phases of participants’ lymphedema journey, 58.4% of patients with cancer-related lymphedema developed symptoms within 12 months of cancer treatment yet, upon further inspection, within the GRA Cohort, some participants developed lymphedema 40 years after treatment. A significantly greater proportion of cancer-related lymphedema patients (61.1%) specified formal lymphedema diagnosis within three months of symptom onset, as compared to non-cancer-related patients (17.3%; Fig. 1A, P < 0.001). In contrast, the majority of non-cancer-related lymphedema patients were diagnosed more than a year later, which was significantly greater than the proportion of cancer-related lymphedema patients who reported this (63.4% vs. 15.9%; Fig. 1A, P < 0.001). In the Interactions Cohort, 50% of cancer-related lymphedema patients were diagnosed by ‘Another Specialist’. Upon further examination, it was noted that 30.4% of the GRA participants were diagnosed by the family physician, or an internist or D.O., whereas 21.4% were diagnosed by a lymphedema specialist. The majority of both cancer-related and non-cancer-related patients reported being the first to notice their lymphedema symptoms (Fig. 1B, 86.3% and 64.9%, respectively). In both cohorts, doctors/healthcare providers were the next most common to first identify patients’ lymphedema (22.4% non-cancer-related group vs. 11.5% cancer-related group), followed by family members or friends (12.7% non-cancer-related group vs. 2.2% cancer-related group). It was more common for these external groups to first identify lymphedema within non-cancer-related lymphedema patients (Fig. 1B, P < 0.001). Finally, it was noted that a greater proportion of cancer-related lymphedema patients only consulted one physician prior to receiving a lymphedema diagnosis (53.2% vs. 19.5%; Fig. 1C, P < 0.001), whereas 80.5% of non-cancer-related lymphedema patients were required to see more than one doctor prior to diagnosis (Fig. 1C, P < 0.001). It was noted that 31.2% of non-cancer-related lymphedema patients saw five or more physicians prior to receiving the lymphedema diagnosis, demonstrating a significant disparity in diagnostic care between lymphedema sub-groups (Fig. 1C).

#### Lymphedema treatment

**Figure 2:**
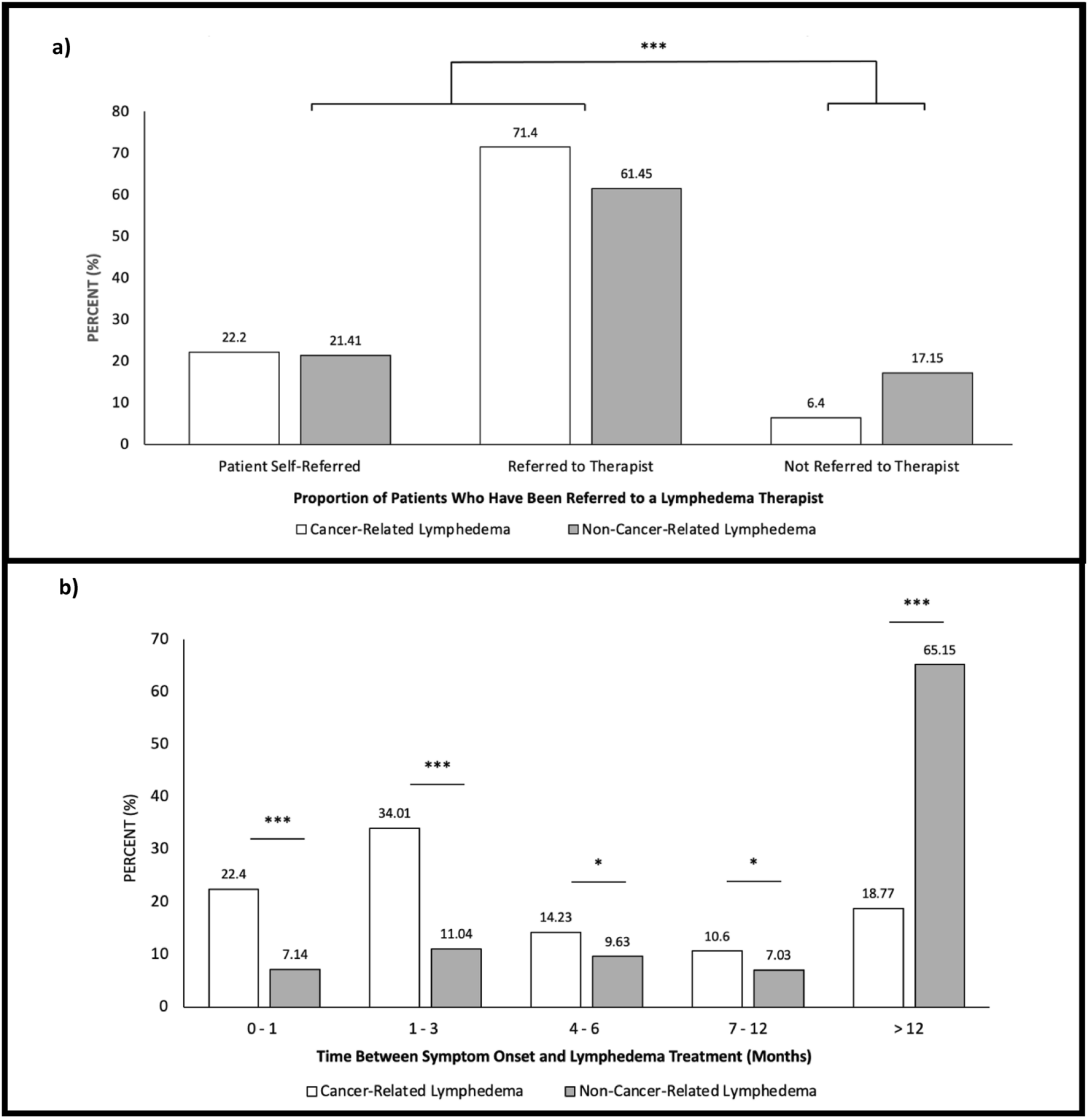
(**a**) Number of participants who were referred to lymphedema therapist or self-referred, two-sample proportions test, ***P < 0.001 (n = 1939) (**b**) Number of months between lymphedema symptom onset and treatment initiation, two-sample proportions test, *P < 0.05, ***P < 0.001, (n = 1923).

The majority of participants, without regard to lymphedema sub-type, reported having been referred to a lymphedema therapist (88.4% vs. 11.6%; Fig. 2A, P < 0.001). These data were echoed within the GRA cohort where more than 80% of patients were receiving lymphedema therapy (P < 0.001). Approximately 22% of participants self-referred in both sub-groups (Fig. 2A). A significantly greater proportion of cancer-related lymphedema patients waited less than one month from symptom onset to lymphedema treatment, when compared to non-cancer-related lymphedema patients; however, this was only representative of 22.4% of the cancer-related cohort (22.4% vs. 7.1%; Fig. 2B, P < 0.001). Approximately 71% of the cancer-related lymphedema participants received treatment within 6 months after initial symptom onset. The majority (65.2%) of non-cancer-related lymphedema patients did not receive treatment until more than one year had elapsed after initial symptom onset, once again demonstrating a marked disparity between lymphatic care provision to non-cancer-related and cancer-related lymphedema patients. Of those receiving treatment for lymphedema, 39.1% of non-cancer-related and 35.9% of cancer-related lymphedema patients reported problems associated with the physical therapy. These challenges included no treatment response (22.1%) and pain (30.7%).

#### Quality of life and healthcare interactions

Despite disparities in patterns of diagnosis and treatment, both cancer-related and non-cancer-related lymphedema patients reported a negative impact of lymphedema on quality of life. Comparisons of sliding scale results showed unison skew towards the ‘devastating’ tail of the scale, with non-cancer-related lymphedema patients reporting significantly worse scores than the cancer-related lymphedema patients (6.7 vs. 6.2, P < 0.001). Furthermore, the mean rating for physician interest was skewed towards ‘uninterested’, indicating lack of medical support perceived by both cancer-related lymphedema and non-cancer-related lymphedema patients (− 3.8 vs. − 4.1, P = 0.1773). Finally, the mean rating for patient satisfaction with their lymphedema diagnosis and treatment was skewed towards ‘dissatisfied’ (2.8 vs. 3.1, P = 0.0037), indicating a need for improved diagnostics and treatment for both cancer-related and non-cancer-related lymphedema.

### Healthcare interactions: cancer-related lymphedema patients

#### Baseline characteristics and pre-oncologic care

The following results relate to cancer-related lymphedema patients only. The majority (65.3%) of patients with cancer-related lymphedema reported both lymphadenectomy and radiation components to the initial cancer treatment (Fig. 1E). Only 2.7% of participants reported having neither. Most patients reported not having had a discussion with their healthcare provider nor receiving educational materials on lymphedema prior to cancer treatment (66.2% and 86.4%, respectively). Furthermore, 90.6% of cancer-related lymphedema patients reported not receiving a prescription for prophylactic compression garments prior to cancer treatment.

#### Peri-treatment and post-treatment lymphedema care

**Figure 3:**
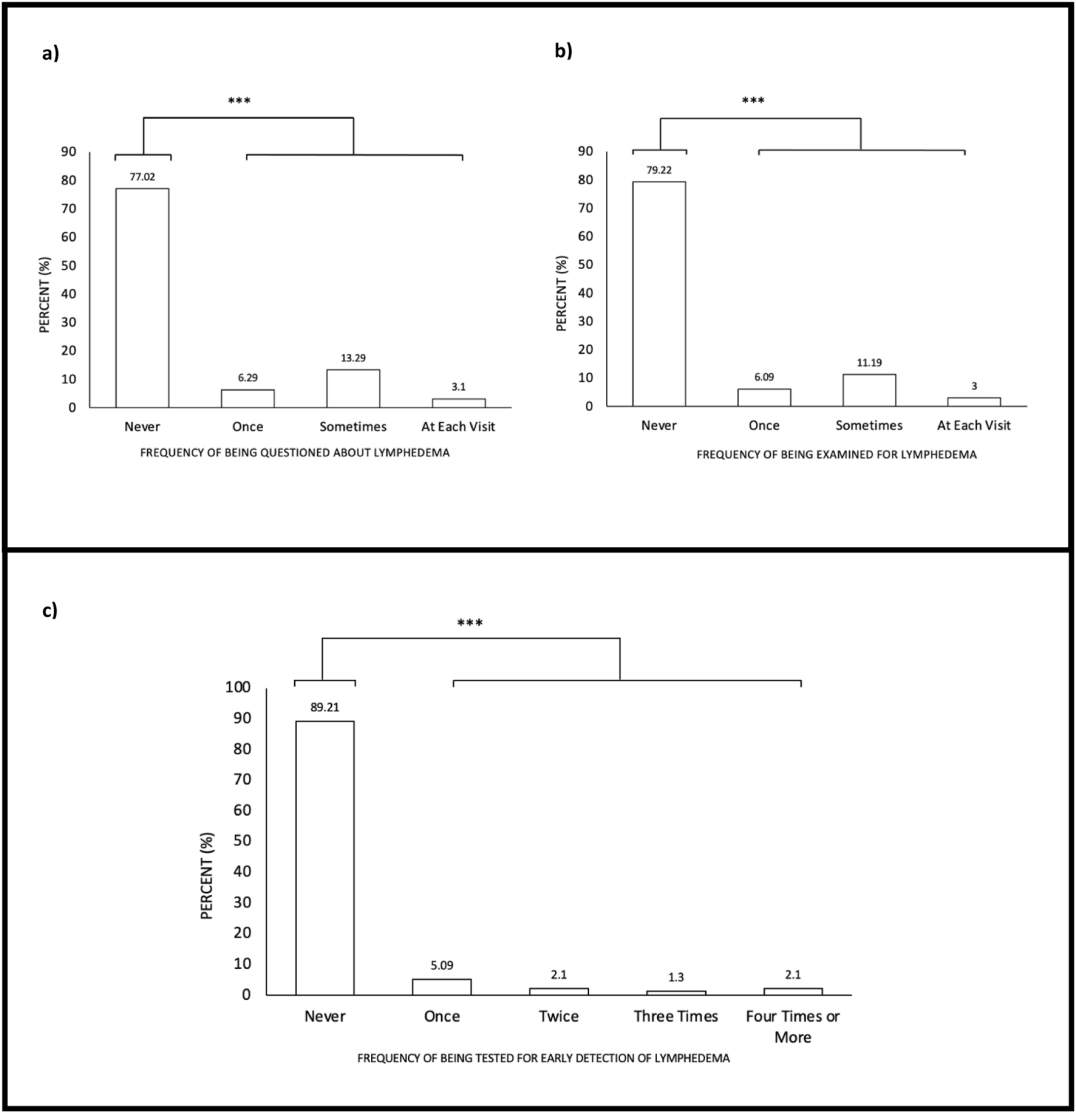
(**a**) Percentage of patients who report having been questioned about lymphedema during cancer treatment, one-sample proportions test ***P<0.01 (n=1001)  (**b**) Percentage of patients who report having been examined for lymphedema during cancer treatment, one-sample proportions test ***P<0.01 (n=1001) (**c**) Percentage of patients who reported being tested for early detection of lymphedema during cancer treatment, one-sample proportions test *** P < 0.001 (n = 1001).

The majority of patients reported never being questioned about lymphedema symptoms or being physically examined for lymphedema during cancer treatment (77.0% and 79.2%, respectively), representing significantly greater proportions than those receiving these forms of lymphatic care during cancer treatment (77.0% vs. 22.7% and 79.2% vs. 20.3%; Fig. 3A,B, P < 0.001). Furthermore, the majority of patients reported never being tested for early detection of lymphedema during their cancer treatment (89.2% vs. 10.6%; Fig. 3C, P < 0.001). However, the majority (58.4%) of participants reported developing symptoms of lymphedema within 12 months of cancer treatment. Interestingly, 50% of participants were diagnosed by a physician other than the cancer surgeon, radiation oncologist, or primary care physician (Fig. 1D). Approximately, 16% of participants reported never receiving a diagnosis from a physician. The majority of cancer-related lymphedema patients reported never being formally tested for lymphedema (57.0%).

## Discussion

**Figure 4:**
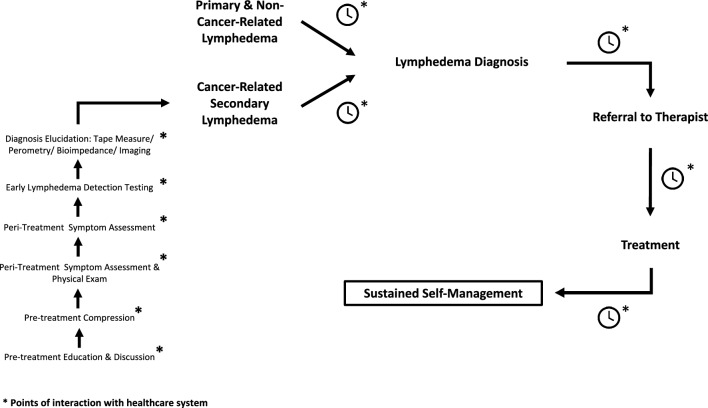
Model of healthcare disparities faced by lymphedema patients and potential points of intervention developed based upon data collection.

This study is the first attempt to formally validate and quantitate widespread experiences and levels of dissatisfaction of lymphedema patients interacting with the medical system. Findings were integrated into a model of healthcare disparities faced by lymphedema patients and potential points of intervention (Fig. 4). Results demonstrated that both cancer-related and non-cancer-related lymphedema patients experienced substantial delays between symptom onset and diagnosis, if any diagnosis was received at all. These findings align with published cohort and qualitative studies that underscore the impact of delayed diagnosis on patient quality of life, particularly in the cancer-related lymphedema population^2,14,15^. Few studies have documented this delay in non-cancer related lymphedema populations. However, our study results indicate these patients experience greater delays between symptom onset and diagnosis than cancer-related lymphedema patients likely due to the lack of recognition of lymphedema as a medical pathology within the general medical community. Although lymphedema patients were the most likely group to identify their first symptoms, healthcare providers also played a pivotal role. This finding emphasizes the need for strong healthcare support networks for patients during the initial stages of disease onset. Within the context of cancer-related lymphedema, patients have frequent interactions with the healthcare system, emphasizing the many points of intervention that already exist in the patient journey for lymphatic education and diagnosis^16,17^. This concept becomes increasingly difficult with non-cancer-related lymphedema patients due to the variability in presentation, time of disease onset, and frequency of patient interactions with the medical system^18,19^. Hence, global education of both generalists and subspecialists on lymphatic diseases is pertinent to improve screening, diagnosis, and treatment of these patients.

Lymphedema treatment was not initiated in the majority of non-cancer-related lymphedema patients until 12 months after symptom onset, representing a vital opportunity to modulate lymphedema progression through the use of various treatment modalities. For instance, in some care models, early consideration of lymphedema surgery is included, but given the nature of data collection within this study, there was insufficient information to include it within our proposed model. Furthermore, non-cancer-related lymphedema patients experienced substantially greater wait times than cancer-related lymphedema patients. This may be due to the unique level of lymphatic education in each medical domain that is likely to entrain such patients and therefore, the knowledge of available treatment networks.

Collectively, participants reported a negative impact of lymphedema on quality of life. Non-cancer-related lymphedema participants reported scores that were significantly more negative than cancer-related lymphedema patients. Non-cancer-related lymphedema patients continue to face medical neglect, possibly due to the fact that there is no uniquely defined medical domain dedicated to the treatment of lymphatic pathologies^1,4,7,20^. These patients also experienced longer waits between symptom onset and diagnosis, as well as diagnosis to treatment^18^. These greater time lapses abandon patients to address burdensome lymphedema symptoms with little to no support from their healthcare providers. Not only does this lead to worsening of physical symptoms, but it also creates a complex dynamic of psychosocial morbidity and uncertainty that influence quality of life^4,21^. Patient satisfaction with the schema of lymphedema diagnosis and treatment was also skewed towards ‘dissatisfied’, without regard to lymphedema subtype. These data demonstrate that no matter the experience of each specific lymphedema sub-group, all patients share this negative view of their lymphatic healthcare. Finally, both groups reported disinterest from their physicians, with the non-cancer-related lymphedema participants reporting a significantly lower level of interest from their physicians than cancer-related lymphedema patients. These data shed light upon an alarming reality that lymphedema is not only poorly understood by the medical community, but it also may not be of interest for healthcare providers who predictably care for lymphatic patients^9,21,22^. Hence, the foundational importance of the lymphatic system must be clearly emphasized prior to educating physicians on lymphatic pathologies since, without physician interest, there will be little uptake of educational material.

The results compiled on cancer-related lymphedema patients demonstrated that the majority of lymphedema patients reported not benefiting from a discussion or exposure to educational materials on lymphedema prior to cancer treatment. In parallel, there was documentation of underutilization of prophylactic compression garment prescription. This lack of education was further perpetuated throughout cancer treatment, where 77–79% of participants reported never being questioned about lymphedema symptoms or being physically examined for its manifestations during cancer therapy. Finally, almost 90% of participants reported never being tested for early detection of lymphedema during cancer treatment. These observations emphasize the potential impact of the many interactions that cancer patients will have within the medical system throughout cancer therapy. It is therefore not surprising that 50% of participants received the lymphedema diagnosis from a physician outside of the cancer or primary care team, despite having developed symptoms within 12 months of cancer treatment. The evidence demonstrates that if lymphedema is not expressly considered during routine visits throughout the cancer therapy continuum, these patients will not receive adequate lymphatic care, with predictable worsening of physical and psychosocial distress (Fig. 4).

This study has its limitations. Demographic data collection was intentionally omitted for the Interactions Cohort. We aimed to maintain HIPAA compliance and yield the largest possible return of full questionnaire submissions. As such, the addition of several questions relating to demographics to the survey was deemed a potential threat to compliance. This choice prevents an assessment of selection bias and generalizability of the Interactions Cohort data. Therefore, we identified the LE&RN global patient registry as a representative subset of the Interactions Cohort participants to obtain demographic and clinical data. Although it is unlikely that we captured exact overlap between cohorts, the use of LE&RN organizational networks for recruitment increased the likelihood that the sample was representative. Uniform and consistent use of diagnostic criteria continues to be a challenge in the context of lymphedema; therefore, self-report was used^23^. Self-report data are susceptible to misclassification bias and therefore, it is possible that participants with other forms of chronic edema were included. Nevertheless, the central role of the lymphatic system in the prevention of chronic edema (without regard to pathogenesis) suggests that this non-differential misclassification may not adversely influence the inferences derived from the collected data^24^.

## Conclusions and implications for care

The findings of this study emphasize the need for future research on the implementation of novel lymphatic healthcare delivery systems and policies across the United States and beyond. We have identified several gaps in the lymphatic care continuum, which serve as potential points of intervention for future healthcare reform research (Fig. 4). It will be critical to study the perspectives of healthcare practitioners on lymphatic care delivery to further elucidate the mechanisms through which these care disruptions operate in order to develop interventions that are optimally informed. This work will provide the foundation for additional research exploring the most efficient areas and methods through which the lymphatic care continuum can be modified and upheld. It is evident lymphedema has a substantial impact upon patient well-being, yet the current healthcare system appears relatively underprepared to deliver lymphatic healthcare. Patients routinely report delays in diagnosis and treatment with predictable adverse consequences to both clinical presentation, natural history, and healthcare system burden. Research and reform are pivotal to the ability to optimize lymphatic education and healthcare delivery.

### Supplementary Information

Supplementary Information.

## Data Availability

De-identified data and a corresponding data dictionary are available directly through the corresponding author upon reasonable request.
